# Microgravity and Musculoskeletal Health: What Strategies Should Be Used for a Great Challenge?

**DOI:** 10.3390/life13071423

**Published:** 2023-06-21

**Authors:** Roberto Bonanni, Ida Cariati, Mario Marini, Umberto Tarantino, Virginia Tancredi

**Affiliations:** 1 Department of Clinical Sciences and Translational Medicine, “Tor Vergata” University of Rome, Via Montpellier 1, 00133 Rome, Italy; roberto.bonanni1288@gmail.com (R.B.); umberto.tarantino@uniroma2.it (U.T.); 2 Department of Systems Medicine, “Tor Vergata” University of Rome, Via Montpellier 1, 00133 Rome, Italy; mario.marini@uniroma2.it (M.M.); tancredi@uniroma2.it (V.T.); 3 Department of Orthopaedics and Traumatology, “Policlinico Tor Vergata” Foundation, Viale Oxford 81, 00133 Rome, Italy; 4 Centre of Space Bio-Medicine, “Tor Vergata” University of Rome, Via Montpellier 1, 00133 Rome, Italy

**Keywords:** microgravity, musculoskeletal system, bone mineral density, muscle atrophy, weightlessness, prevention, nutrition, antioxidant, exercise, spaceflight

## Abstract

Space colonization represents the most insidious challenge for mankind, as numerous obstacles affect the success of space missions. Specifically, the absence of gravitational forces leads to systemic physiological alterations, with particular emphasis on the musculoskeletal system. Indeed, astronauts exposed to spaceflight are known to report a significant impairment of bone microarchitecture and muscle mass, conditions clinically defined as osteoporosis and sarcopenia. In this context, space medicine assumes a crucial position, as the development of strategies to prevent and/or counteract weightlessness-induced alterations appears to be necessary. Furthermore, the opportunity to study the biological effects induced by weightlessness could provide valuable information regarding adaptations to spaceflight and suggest potential treatments that can preserve musculoskeletal health under microgravity conditions. Noteworthy, improving knowledge about the latest scientific findings in this field of research is crucial, as is thoroughly investigating the mechanisms underlying biological adaptations to microgravity and searching for innovative solutions to counter spaceflight-induced damage. Therefore, this narrative study review, performed using the MEDLINE and Google Scholar databases, aims to summarize the most recent evidence regarding the effects of real and simulated microgravity on the musculoskeletal system and to discuss the effectiveness of the main defence strategies used in both real and experimental settings.

## 1. Introduction

Life on Earth developed under the influence of gravity, so organisms are constantly subjected to loading forces that provide a range of mechanical stimuli essential for the functioning of many physiological systems [1]. The lack of gravity and the resulting loss of mechanical stimulation of cells and tissues are essential characteristics of space, which is considered a hostile environment [2]. The effects induced by the microgravity that characterizes the space environment can be reproduced on Earth using tools and/or strategy, such as the clinostat, random positioning machine, and mechanical limb unloading [3].

The first investigations into musculoskeletal disorders during spaceflight were conducted in parallel by the Soviet Union and the United States. Particularly, Russian scientists discovered many effects of microgravity on cosmonauts during the 1960s, showing changes in biological properties with decreasing gravitational force and emphasizing the close relationship between physical strength and biological function [4]. Indeed, cells exposed to simulated microgravity can be profoundly affected by physical changes, such as loss of gravity-dependent convection, negligible hydrodynamic shear, and lack of sedimentation [5,6], which can significantly alter enzymatic, genetic, and epigenetic processes, causing changes in the form, function, and behavior of cells and tissues [7,8,9]. These alterations result in loss of bone and muscle mass [10,11,12], cardiovascular dysfunction [13,14], impaired fracture healing [15] and wound repair processes [16], as well as impaired immune response [17,18] and vestibular disorders in the ears [19]. Not surprisingly, such damage was found in all crew members of the Gemini Programme, developed by the United States during 1963–1966, in association with loss of bone calcium and muscle nitrogen, reduced post-flight exercise capacity, erythrocyte mass loss, and post-flight orthostatic intolerance [20]. Interestingly, in 1976, Michel et al. observed that bone mass loss in astronauts, in association with calcium, nitrogen, and phosphorus imbalance, occurred at a similar rate to that found in bed rest studies, producing a reduction in leg volume attributable to muscle atrophy and fluid loss [21].

Noteworthy, physical deterioration has been proposed to be counteracted using certain countermeasures, including regular exercise on treadmills and cyclettes and the use of special suits during working hours to stress the skeletal muscles, suggesting this strategy as a means of preventing the alterations induced by weightlessness [22]. Unfortunately, this strategy seems to be insufficient to completely preserve bodily functions, as the consequences of weightlessness persist even after the return to Earth’s gravity, significantly reducing work efficiency and quality of life [23]. Therefore, since space exploration is the new frontier of mankind, and the identification of defence strategies capable of counteracting the effects of being in space is necessary, laying the foundations also for the treatment of certain diseases induced by allurement and/or prolonged sedentariness, such as osteoporosis and sarcopenia.

Based on this evidence, the aim of our narrative review was to (i) describe the biological and molecular alterations of microgravity on the musculoskeletal system, (ii) list possible defence strategies to counteract the impact of weightlessness on bone and muscle tissue, and (iii) emphasize how such countermeasures may represent valid therapeutic options to counteract the musculoskeletal alterations found in both astronauts and elderly subjects forced into a sedentary lifestyle.

## 2. The Literature Search Strategy

A non-systematic search strategy was adopted for this narrative review, which allowed the selection of 151 scientific articles on the impact of microgravity on the musculoskeletal system, with a focus on potential defence strategies to counteract the bone and muscle mass loss induced by weightlessness. Articles of interest published between 1945 (start date) and 2023 were selected via the MEDLINE and Google Scholar bibliographic databases. The search strategy was based on the use of the following combinations of medical subject headings (MeHS) and keywords: (bone) OR (muscle) OR (musculoskeletal system) OR (bone mass) OR (muscle mass) OR (bone mineral density) OR (muscle atrophy) OR (osteoporosis) OR (sarcopenia) OR (preventive strategies) OR (nutrition) OR (antioxidant) OR (exercise) OR (myostatin) OR (irisin) AND (microgravity). For each combination listed, the keyword “microgravity” was replaced with the terms “weightlessness”, “simulated microgravity”, and “spaceflight”. Other articles consistent with the topic were selected independently of the search strategy and included in our manuscript.

The results included in vitro and in vivo experimental studies, narrative reviews, systematic reviews, meta-analyses, clinical trials, and randomized controlled trials to provide a comprehensive overview. Two researchers analyzed all research results by defining their relevance to the topic, while a third researcher resolved any disagreements during the article selection process. Finally, two other authors performed a further check of the selected articles, confirming their validity and clarifying any doubts.

The search process was performed on a worldwide basis, without excluding specific geographical areas or different ethnic groups. Language filters were applied to the list of results to eliminate non-English language articles.

## 3. Physiological Consequences of Microgravity: A Focus on the Musculoskeletal System

**Figure 1:**
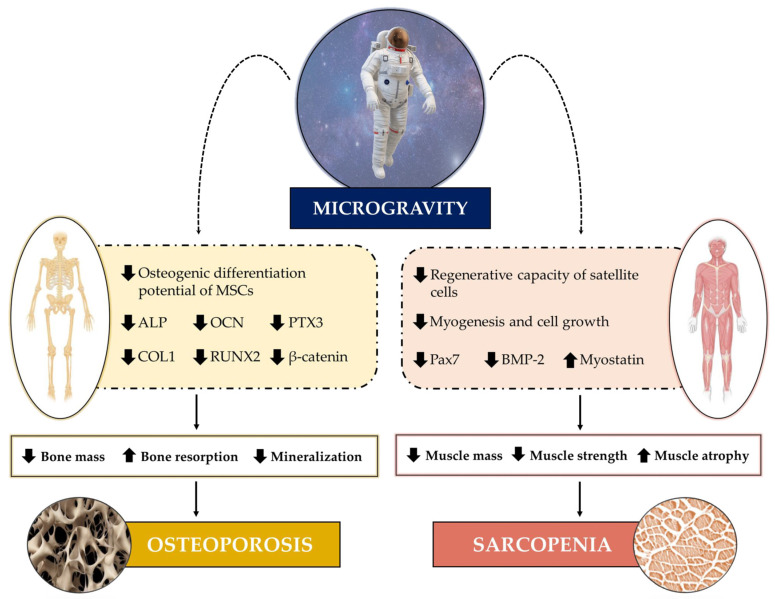
Impact of microgravity on the musculoskeletal system. Microgravity exposure affects bone metabolism, reducing the osteogenic differentiation potential of mesenchymal stem cells (MSCs), as well as the expression of several bone biomarkers, including alkaline phosphatase (ALP), osteocalcin (OCN), pentraxin 3 (PTX3) collagen type I (COL1), runt-related transcription factor 2 (RUNX2), and β-catenin. Consequently, bone mass loss, increased bone resorption, and reduced mineralization occur, all of which promote the onset of osteoporosis. Muscle metabolism is also affected by microgravity exposure. A reduction in the regenerative capacity of satellite cells, a reduction in myogenesis and cell growth, as well as a reduction in the expression of paired box 7 (Pax7) and bone morphogenetic protein 2 (BMP-2) and an increase in the expression of myostatin, are among the main alterations in muscle related to the onset of sarcopenia.

The impact of weightlessness can be seen on numerous organs and apparatuses, as short and long-duration spaceflights are known to induce a wide variety of physiological stresses that result in the impairment of their structural and functional integrity [24]. Much evidence has indicated, as an underlying mechanism, an imbalance in the distribution of interstitial fluid that, in the absence of gravity, is reduced by about 40% in the legs and shifts to the head, altering the pressure of the different anatomical districts [25]. This pressure variation is responsible for several physiological alterations that are commonly found in astronauts exposed to spaceflight, such as swelling of the forehead and facial tissue [26], spaceflight-associated neuro-ocular syndrome (SANS) caused by optic disc oedema [27], as well as increased kidney stone formation [28]. Undoubtedly, damage to the musculoskeletal system is among the most obvious manifestations of microgravity (Figure 1) and is reminiscent of the pathophysiological changes that affect bones and muscles during aging or during prolonged periods of bed rest and/or sedentary activity, such as osteoporosis and sarcopenia [29]. Indeed, simulated microgravity exposure is known to inhibit the differentiation and number of osteoblasts [30,31,32], as well as to induce skeletal muscle cell atrophy [33], confirming its marked impact on musculoskeletal health. Hence, the need to investigate musculoskeletal adaptations to microgravity to fully understand the biological mechanisms responsible for bone and muscle cell damage occurring in both astronauts and patients with osteoporosis and sarcopenia.

### 3.1. Microgravity Effects on Muscle

Skeletal muscles perform several functions that are essential for human life, as they not only provide the strength needed to counteract gravity and maintain posture, but also constitute a storehouse of important substrates, such as amino acids and carbohydrates, that are crucial for heat and energy production [34]. Exposure to real or simulated microgravity is known to result in a significant loss of muscle mass and strength, leading to muscle atrophy, changes in the composition and gene expression of muscle fibres, and a reduction in the regenerative capacity of satellite cells [35]. Undoubtedly, weightlessness-induced loss of muscle mass has been a medical and physiological concern, starting from the earliest space missions [4]. However, despite the important knowledge acquired on the physiological changes induced by spaceflight and the progress of research in this field, the molecular mechanisms responsible for muscle atrophy, as well as the effectiveness of possible countermeasures taken, still need to be investigated in depth.

In this context, experiments conducted on animal models subjected to spaceflight or lower limb unloading have shown that postural muscles, which generally contain a higher percentage of slow fibres, are more prone to atrophy than non-postural muscles [36,37,38]. Similar results have also been found for human skeletal muscles, as the effects of simulated microgravity appear to be more pronounced in antigravity muscles, i.e., those that play a postural role, such as the soleus, gastrocnemius, and quadriceps muscles [39,40,41]. In fact, during spaceflight or in a microgravity environment, the antigravity muscles are subject to atrophy as, in the absence of a gravitational field, the need to support the body is lost [42]. In this regard, Akima et al. investigated the volume changes of the extensor, flexor, and plantar flexor knee muscle groups of three crew members, identified as subjects A, B, and C, before and after a spaceflight of approximately 9, 15, and 16 days [43]. The greatest decrease in knee extensor muscle volume was found four days after the flight for subjects A (−15.4%) and C (−11.6%) and one day after the flight for subject B (−5.6%). Interestingly, the volume of the extensor and flexor muscles of the knee of subjects B and C almost recovered 21 and 30 days after landing, respectively, in contrast to subject A, who took between 30 and 120 days for full recovery. Similarly, a significant reduction in the volume of the plantar flexor muscles was observed, while full recovery took between 30 and 120 days, suggesting that alterations in muscle volume vary widely depending on the individual and the muscle group considered [43]. Overall, most studies have shown that the extensor muscles are more affected by microgravity than their antagonists, the flexors. For example, Widrick et al. observed that flexor fibres are less affected than extensor fibres after a 17-day spaceflight [44], although it has been reported that skeletal muscles are similarly affected by microgravity for long duration missions [39].

The devastating effects of microgravity on skeletal muscle may depend on the impaired maintenance of the progenitor stem cell pool. In this context, Hosoyama et al. exposed a cell culture model of fluctuating spherical aggregation of progenitor stem cells to clinostatic rotation for two weeks, observing a reduction in paired box 7 (Pax7), a transcription factor that plays a critical role in myogenesis, suggesting a dependence of muscle atrophy under microgravity conditions on stem cell pool depletion [45]. Similar results were obtained by Tarantino and colleagues, who recently investigated the role of myostatin, the most famous negative regulator of muscle growth, and bone morphogenetic protein 2 (BMP-2), in the response of human satellite cells isolated from patients undergoing hip arthroplasty for high-energy fracture, osteoarthritis, or osteoporosis exposed to random positioning machine (RPM) for three days [46]. Interestingly, increased myotube formation and increased BMP-2 expression were detected in all experimental groups already in the very early stages of RPM exposure. However, prolonged exposure to microgravity induced significant changes in myostatin expression, concomitant with the degeneration of satellite cells and myotubes, suggesting that the altered BMP-2/myostatin ratio may be responsible for the muscle atrophy and impaired regenerative potential that occurs under simulated microgravity conditions [46].

Interestingly, altered protein turnover has been suggested as the molecular mechanism responsible for load-free muscle atrophy. Indeed, hypokinesia and hypodynamia in rats subjected to hind limb unloading have long been known to cause a rapid reduction in protein synthesis, such as that observed in subjects exposed to prolonged bed rest or spaceflight [47,48,49]. This effect could depend on an increase in the dephosphorylation events of the phosphoinositide 3-kinase (PI3K)/protein kinase B (Akt) pathway, which would lead to its inactivation [50,51]. Indeed, the generation of mice lacking Akt or the insulin-like growth factor (IGF-1) receptor results in a phenotype characterized by a severe growth deficit and significant muscle atrophy, highlighting a crucial role of the PI3K/Akt/mammalian target of rapamycin (mTOR) pathway in the muscle growth regulation [52]. Furthermore, inhibition of this pathway in muscle cells is known to induce down-regulation of most IGF-1-regulated genes, confirming its involvement in the protein synthesis modulation. Indeed, intense muscular stimulation through maximal resistance exercise or with anabolic agents, such as IGF-1, results in the PI3K/Akt pathway activation and the consequent phosphorylation of mTOR. This results in the activation of specific downstream kinases, as well as the inactivation of repressors of protein synthesis, promoting an increase in the synthesis of muscle proteins that lead to growth and hypertrophy [53].

On the other hand, in addition to the reduction in protein synthesis, the prolonged absence of loading also leads to the activation of proteolytic systems that cause protein degradation, favoring muscle wasting [54]. Particularly, the ubiquitin–proteasome system has been proposed to be most involved in disuse muscle atrophy, as several mRNAs encoding proteins involved in this system are increased in muscle atrophy induced by hind limb unloading and spaceflight. These include mRNAs coding for muscle RING-finger protein-1 (MuRF1) and muscle atrophy F-box (MAFbx)/atrogin-1, two muscle-specific classes of ubiquitin-ligase (E3), an enzyme that recognizes multiple target protein substrates by regulating proteolysis [54]. Noteworthy, the expression of these muscle-specific ubiquitin ligases is regulated by fork head box O (FOXO) transcription factors, whose activity is under the control of the IGF-1-PI3K-Akt pathway [55]. Specifically, under unloaded conditions, Akt does not phosphorylate FOXO, which translocate into the nucleus and up-regulates the expression of ubiquitin ligases, highlighting the PI3K/Akt pathway involvement in both the reduction of protein synthesis and the increase in degradation due to unloading [56].

A further mechanism suggested to be involved in disuse muscle atrophy is increased oxidative stress, which is one of the main triggers of the imbalance between protein synthesis and degradation that leads to muscle atrophy [57,58,59]. Indeed, increased reactive oxygen species (ROS) promote the expression of proteins involved in proteolytic pathways, as well as proteases calpain and caspase-3, promoting protein degradation and apoptosis [58]. Interestingly, calcium is a known important regulator of calpain activation, suggesting its role in disuse-induced protein degradation [60]. In this context, Matsumoto et al. observed the calcium-mediated activation of the ubiquitous calpain calpain1 and calpain2 in rats subjected to upper limb unloading [61]. The deregulation of intracellular calcium levels could be responsible for the calpain activation and, consequently, the increase in protein degradation, as demonstrated by the alterations in the release and reabsorption of calcium from the sarcoplasmic reticulum observed after six days of spaceflight [62,63].

Finally, mitochondrial dysfunction appears to play a crucial role in spaceflight-induced cellular and tissue alterations, leading to an impairment of cellular respiration processes, adenosine triphosphate (ATP) production, oxidative phosphorylation, and mitochondrial gene expression [64]. In this context, peroxisome proliferator-activated receptor γ coactivator 1α (PGC-1α), a promoter of mitochondrial biogenesis, is known to play a central role in mitochondrial alterations and muscle atrophy, as a reduced expression of PGC-1α, concomitant with significant muscle atrophy, has been observed in both human and mouse discharge models [65,66]. Overall, metabolic alterations affecting skeletal muscle under microgravity or no-load conditions result in reduced force-generating capacity [67]. This is mainly due to the decrease in the size of individual muscle fibres, as demonstrated by muscle biopsies from astronauts exposed to spaceflight, resulting in an overall reduction in specific force [68,69].

Unfortunately, existing data on the effect of spaceflight on skeletal muscles are rather heterogeneous, probably due to numerous factors, such as age, fitness, and nutritional status prior to flight, as well as the adoption of countermeasures to minimize the impact of weightlessness [35]. Furthermore, discordant data have also been reported on the type of muscle fibres that are primarily affected by microgravity. Indeed, a preferential atrophy of type I, slow, and fatigue-resistant muscle fibres has been observed in rats maintained under simulated microgravity [10]. On the other hand, in human skeletal muscles, muscle atrophy seems to affect both types of fibres equally, although some authors have reported greater atrophy in type II fibres, in conjunction with a significant reduction in the cross-sectional area of the vastus lateralis muscle [70].

### 3.2. Microgravity Effects on Bones

Bones are complex, constantly evolving tissues that provide the anchoring site for muscles and respond to changes in load, while also regulating mineral homeostasis through their involvement in metabolic pathways [71]. Indeed, bone is known to be a metabolically active organ that undergoes continuous remodelling throughout life through the phenomena of bone formation and resorption [72]. This process is operated by two types of cells that populate bone tissue: osteoblasts, which are responsible for the formation of new bone tissue, and osteoclasts, which instead drive resorption [73]. For the proper maintenance of homeostasis, the activity of these two cell types must be finely regulated, as deregulation of their activity could lead to the onset of metabolic bone diseases, such as osteoporosis [74]. 

For the activity of osteoblasts and osteoclasts to be in balance, thus maintaining the optimal microarchitecture of bone tissue, the mechanical stresses that characterize gravitational fields are necessary. However, in a microgravity environment, the absence of such mechanical stresses results in a rapid decoupling between formation and resorption, leading to a reduction in bone mass and promoting the onset of osteoporosis [75]. This condition, which has been compared to that observed in post-menopausal women, is known as spaceflight osteopenia, and it is manifested in astronauts by the bone mass loss in the proximal femur [76]. Therefore, bone mass loss due to weightlessness is a critical issue that needs substantial investigation at the molecular and cellular level. In recent years, many studies have been conducted to identify the biological changes induced by real or simulated microgravity that occur in mesenchymal stem cells, osteoblasts, and osteoclasts.

#### 3.2.1. Mesenchymal Stem Cells (MSCs)

MSCs are multipotent stem cells present in the bone marrow and are important for their ability to differentiate into chondrocytes, osteoblasts, and adipocytes [77]. However, under simulated microgravity conditions, their differentiation potential may alter, favoring the maintenance of an undifferentiated state [78]. In this context, Liu and colleagues studied mitochondrial oxidative phosphorylation by assessing PGC-1α levels in MSCs under osteogenic and simulated microgravity conditions [79]. A significant inhibition of osteogenic differentiation and oxidative phosphorylation of MSCs was detected, concomitant with a reduction in the expression of sirtuin 1 (SRT1), an NAD^+^-dependent protein deacetylase that regulates energy metabolism. Interestingly, the use of resveratrol, a potent antioxidant that activates SRT1, resulted in SRT1 overexpression and preserved oxidative phosphorylation and osteogenic differentiation, suggesting that SRT1 upregulation may counteract the damaging effects of simulated microgravity [79].

The osteogenic differentiation potential loss could be caused precisely by the absence of mechanical stress, as microgravity exposure, as well as lack of physical activity due to spinal and brain injury, can significantly reduce mechanical stress and favour the adipogenic fate of MSCs, rather than the osteogenic one [80,81]. Although the mechanism that promotes the osteogenic-adipogenic transition in the absence of loading has not yet been fully elucidated, some evidence has suggested that weightlessness might influence the differentiation potential of MSCs by downregulating mitogen-activated protein kinase (MAPK) and increasing the peroxisome proliferator-activated receptor gamma (PPAR-γ) expression [82]. The cytoskeleton is also strongly influenced by signals from the extracellular microenvironment, being able to transduce the mechanical signals that cause deformation of the extracellular matrix [83,84]. However, simulated microgravity could result in the collapse of the actin microfilaments of the cytoskeleton, causing cell cycle arrest in the G0/G1 phase [85].

#### 3.2.2. Osteoblasts and Osteoclasts

Osteoblasts are responsible for new tissue formation, playing a key role in maintaining bone homeostasis [86]. The effect of simulated microgravity on these cells has been known for some time. As early as 1997, Carmeliet et al. observed a significant reduction in alkaline phosphatase (ALP) activity and osteocalcin (OCN) expression in osteoblasts exposed to simulated microgravity [30]. Similarly, a five-day RPM exposure of the human osteosarcoma cell line SAOS-2 under osteogenic conditions caused a significant reduction in mineralization, calcifying nodules’ presence and pentraxin 3 (PTX3) expression, an important regulator of bone metabolism [87]. Furthermore, weightlessness has been suggested to inhibit the differentiation of osteoprogenitor cells into mature osteoblasts and reduce the osteogenic potential of bone marrow MSCs [88,89]. Overall, microgravity-induced loss of bone mass could be attributed to impaired activity, proliferation, and differentiation of osteoblasts, which would become less responsive to bone-related factors present in the microenvironment [90]. Alternatively, such physiological adaptation to microgravity could depend on cytoskeletal alterations that occur in osteoblasts in the absence of mechanical stimuli [91], the cytoskeleton being closely associated with nuclear morphology and function [92].

Noteworthy, simulated microgravity exposure is known to damage the actin microfilaments of osteoblasts, leading to a deficit in bone formation [93,94]. In this regard, Fan et al. recently observed that the osteoblast cell line MC3T3-E1 exposed to RPM for three days showed marked structural alteration of the cytoskeleton, reduced formation of focal adhesions, as well as significant down-regulation of focal adhesion kinase (FAK) signalling, a tyrosine kinase that regulates osteoblastic differentiation and bone regeneration [95]. Furthermore, simulated microgravity exposure dramatically reduced the expression of β-catenin and markers of osteoblastic maturation, such as BMP-2 and collagen type I (COL1), as well as inhibiting ALP activity and mineralization. Interestingly, treatment with cytotoxic necrotizing factor-1 (CNF1), which acts as an FAK activator, counteracted the deleterious effects of RPM exposure, suggesting FAK as a potential therapeutic target to prevent weightlessness-induced bone loss [95].

Although the mechanisms underlying weightlessness-induced bone loss have not yet been fully elucidated, altered calcium metabolism could explain the demineralizing effects of space missions [96]. Indeed, in weightlessness, the excessive release of calcium by bone tissue is responsible for the suppression of parathyroid hormone (PTH) and circulating 1,25-dihydroxyvitamin D (1,25(OH)2D), resulting in reduced intestinal calcium absorption that leads to muscle wasting and the onset of osteoporosis [97,98,99]. In this regard, Hu and colleagues found a significant reduction in osteoblastic differentiation and mineralized nodule formation in MC3T3-E1 murine pre-osteoblasts exposed to RPM for 24 h, in association with reduced expression of some important markers of osteoblastic differentiation, such as runt-related transcription factor 2 (RUNX2), OCN and COL1 [100]. In addition, the inhibitory effects of microgravity on osteoblast function were recently confirmed by Braveboy-Wagner and Lelkes, who observed that exposure of 7F2 osteoblasts to various levels of simulated partial gravity resulted in a significant gravity-dependent inhibition of short-term (six days) ALP proliferation and activity and long-term (twenty-one days) mineralization, suggesting a close association between impaired cell function and levels of simulated partial gravity [101].

Noteworthy, microgravity-induced loss of bone mass could also be caused by an alteration of osteoblast proliferation and apoptosis processes. In this context, Bucaro et al. observed in clinostat-exposed MC3T3-E1 cells a significant down-regulation of the anti-apoptotic protein Bcl-2 and Akt, key markers of cell proliferation and survival [75]. Subsequently, Dai and colleagues also confirmed the involvement of Akt in the response of rat bone marrow mesenchymal stem cells exposed to three days of clinorotation, confirming the alteration of proliferative and apoptotic processes among the main mechanisms responsible for the weightlessness-induced loss of bone mass [85].

Finally, the impairment of osteoblastic activity under microgravity conditions has been correlated with an increase in osteoclastogenesis [102,103]. In this respect, Tamma et al. studied the differentiation of osteoclast precursors, cultured on slices of devitalized bovine bone for four days under microgravity conditions inside bioreactors with a perfusion system, into mature osteoclasts [104]. Interestingly, a significant increase in the expression of genes involved in osteoclast maturation and activity was observed, in association with an increase in bone resorption demonstrated by an increased release of collagen telopeptides, suggesting osteoclasts and their precursors as direct targets of microgravity [104]. These findings were confirmed by Sambandam and colleagues, who found over-expression of growth factors in pre-osteoclasts exposed to simulated microgravity, resulting in increased bone resorption [105]. In addition, nano-CT scans and measurements of tartrate-resistant acid phosphatase (TRAP), an osteoclast-specific gene, on C57BL/6 mice exposed to spaceflight for 15 days aboard the space shuttle ST-131, showed marked bone loss and increased osteoclast numbers, confirming the effect of microgravity on bone resorption [106]. Similarly, Chatani and colleagues, aboard two ISS flights, used double transgenic fish, with a TRAP promoter linked to a green fluorescent protein and a metalloproteinase 9 promoter linked to a red fluorescent protein, to identify osteoclasts and assess their activity by RNA extraction and transcriptome analysis. Interestingly, a significant up-regulation of osteoclast activity was observed on days four and six of spaceflight, concomitantly with an increased expression of osteoclast-specific genes after the second day of flight [107].

## 4. Can We Resist Microgravity?

**Figure 2:**
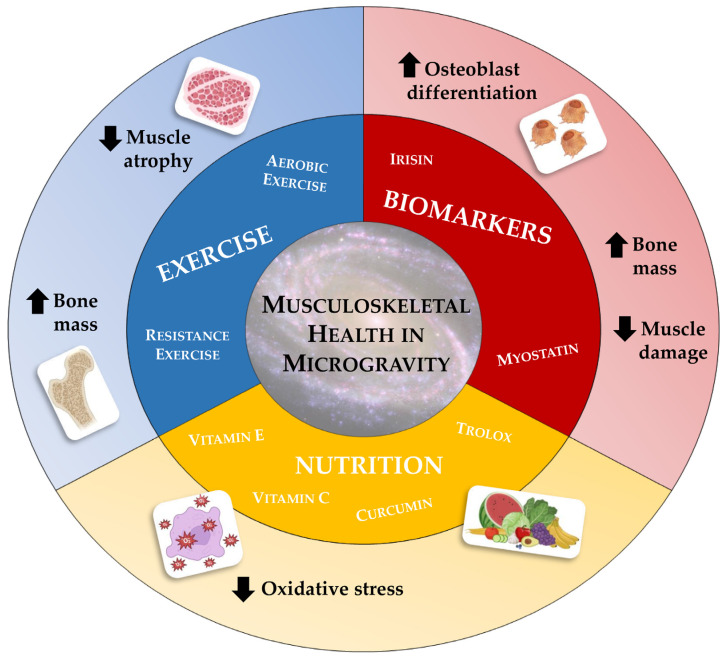
Potential strategies to prevent and/or counteract weightlessness-induced damage to the musculoskeletal system. The most widely used prevention strategies to preserve musculoskeletal health in microgravity include an appropriate nutritional approach and the antioxidants administration, such as vitamin E, vitamin C, curcumin, and trolox, to reduce oxidative stress, in combination with regular exercise, both aerobic and endurance, to increase bone mass and reduce muscle atrophy. In addition, the development of experimental drug therapies, such as the use of anti-myostatin antibodies and recombinant irisin, are among the most innovative choices on which research has recently focused.

In the near future, humanity is about to colonize space and, with it, satellites and planets. This momentous event will bring about a radical change in the way human beings are accustomed to living, necessitating adaptation to hitherto unknown living conditions. As mentioned above, prolonged exposure to very low gravitational forces will lead to unavoidable physiological changes, which will require timely and effective management and preventive action. For this reason, the concept of space medicine must be explored, highlighting the need to develop strategies to prevent and/or counteract physiological alterations induced by weightlessness [108]. In the area of musculoskeletal alterations, numerous options have been explored (Figure 2), although scientific research is still far from identifying an elixir capable of opposing the devastating effects of microgravity.

### 4.1. Nutrition and Antioxidants

Nutrition plays a key role in space travel, from providing all the nutrients to meet the body’s metabolic needs and requirements, to enhancing the individual’s emotional wellbeing. Furthermore, adequate nutrition is crucial to compensate for some of the negative effects of weightlessness, such as oxidative stress and bone and muscle mass loss [109]. For this reason, the World Health Organization (WHO) has established specific nutritional requirements for spaceflight, based on the daily needs of people on Earth. Particularly, the recommended intake of key macronutrients, such as proteins, lipids, and carbohydrates, should also be combined with a constant vitamin intake, given their antioxidant power [110]. Not surprisingly, the ROS generation and the resulting oxidative stress are among the main contributors to the bone mass loss that occurs in microgravity conditions [111]. In this regard, Smith and colleagues found a 32% increase in the urinary concentration of 8-hydroxy-2′-deoxyguanosine (8-OHdG) during long-duration spaceflight, while the concentration of superoxide dismutase was significantly reduced after flight, suggesting potential damage to cellular and nuclear structures as a consequence of weightlessness [112]. In agreement, Rai et al. observed a close association between prolonged exposure to microgravity and increases in specific oxidative stress markers, such as 8-OhdG and malondialdehyde, confirming that mechanical exhaust promotes ROS formation and that this effect is more pronounced in long duration flights and persists for several weeks after the end of the mission [113]. Similar results were observed in mouse models subjected to limb unloading, in which an increase in intracellular levels of ROS, 8-OhdG, and 4-hydroxynonenel (4-HNE) was detected at the same time as significant bone loss caused by reduced osteoblastic capacity [114]. Interestingly, daily administration of an antioxidant, vitamin C, significantly attenuated bone loss during mechanical unloading, preserving bone morphometric parameters, such as bone volume (BV/TV), trabecular thickness (Tb.Th), trabecular number (Tb.N), and trabecular separation at optimal values [114]. Similarly, Xin and colleagues investigated the efficacy of treatment with 1,7-bis(4-hydroxy-3-methoxyphenyl)-1,6-heptadiene-3,5-dione (curcumin), a known antioxidant that reduces free radicals and promotes the expression of various cytoprotective and antioxidant proteins, in counteracting bone loss in rats exposed to hind limb suspension for six weeks. Surprisingly, curcumin treatment proved to be an effective countermeasure to inhibit ROS formation induced by mechanical unloading, enhancing osteoblastic differentiation, and attenuating osteoclastogenesis [115,116]. The extraordinary power of antioxidants was recently confirmed by Morabito et al., who demonstrated how treatment with 6-hydroxy-2,5,7,8-tetramethylchroman-2-carboxylic acid (trolox), a water-soluble analogue of vitamin E, counteracted oxidative damage induced by prolonged exposure to RPM in a murine osteoblast cell line, while also preserving cytoskeleton architecture and restoring intracellular ROS and calcium levels [117].

The involvement of oxidative stress has also been demonstrated in muscle atrophy induced by mechanical exhaustion. In this context, although some antioxidant cocktails have proven ineffective [118,119], greater success has been demonstrated for the overexpression of catalase [120], mitochondria-targeted antioxidants [121], and administration of a mimetic of superoxide dismutase and catalase (EUK-134) [122,123]. Interestingly, Lawler et al. have recently shown that the oxidative stress response and atrophy of skeletal muscle fibres occurring under simulated microgravity conditions can be mitigated by inhibition of the pro-oxidant enzyme NAPDH oxidase-2 (Nox2), a membrane oxidoreductase that produces ROS in response to skeletal muscle contractions and stretch [124]. In agreement with other studies, manganese-dependent superoxide dismutase (MnSOD), an antioxidant enzyme, was also down-regulated with mechanical unloading, in association with the nuclear transcription factor erythroid-2 (Nrf2), providing protection against muscle wasting and oxidative stress typical of the sarcopenic condition [125,126]. Finally, treatment of rats with the fat-soluble antioxidant vitamin E promoted an improvement of muscle wasting atrophy by approximately 20 per cent, just as intramuscular injection of the flavonoid quercetin into the gastrocnemius muscle was observed to effectively prevent muscle weight loss in rats subjected to hind limb unloading [127,128].

### 4.2. Exercise

The benefits of exercise on musculoskeletal health are well known and widely documented. Indeed, regular exercise preserves the function and structure of both bone and muscle tissue, preventing the onset of osteoporosis and sarcopenia [129,130]. Unfortunately, the beneficial effects of exercise on the musculoskeletal system under load-free conditions appear to be only partial, as they do not fully restore bone and muscle mass. In this context, Norman et al. found a complete preservation of bone mineral density (BMD) in rats subjected to limb unloading and treadmill exercise, while their muscle mass was significantly lower than that of controls, indicating a partial beneficial effect of exercise in counteracting weightlessness-induced damage [131]. 

Already during the first space missions, the introduction of regular exercise, increased exercise duration and improved equipment used were observed to mitigate, but not prevent, musculoskeletal changes [132]. This is also true for the first missions to the international space station (ISS), as daily exercise using a treadmill, cycle ergometer, and advanced resistance exercise device (ARED) was beneficial, but not sufficient, to prevent bone and muscle decline in astronauts [133]. In this context, Gopalakrishnan et al. quantified the changes in muscle volume, strength, and endurance of crew members on the ISS, noting a general loss of concentric strength in all lower limb muscle groups, despite the exercise programme [134]. Subsequently, Fitts and colleagues investigated the effects of no-load and exercise countermeasures on anaerobic and aerobic enzyme activity and glycogen and lipid content in the slow and fast fibres of the soleus and gastrocnemius muscles in nine ISS crew members for six months. Post-flight results showed that treadmill running partially protected against muscle atrophy, as well as increasing the muscle’s aerobic enzyme activity [135]. Other evidence subsequently suggested that a combination of resistance training and a mix of high-intensity interval and continuous aerobic training, in association with the use of innovative and appropriate equipment, could be a viable countermeasure in counteracting the loss of bone mass and muscle strength caused by mechanical unloading, although not offering complete prevention [136]. More recently, Gabel and colleagues investigated the effects on bone microarchitecture, density, and strength of the distal tibia and radius in 17 astronauts exposed to spaceflight, looking for possible correlations between mission duration, bone markers, and pre-flight and in-flight exercise on bone morphological changes [137]. Noteworthy, individuals with high pre-flight bone turnover were more sensitive to the negative effects of offloading in microgravity conditions, while a higher probability of preserving bone strength and tibia trabecular bone was found in crew members who increased the volume of in-flight endurance training compared to pre-flight. Overall, these results highlight that pre-flight bone turnover markers and exercise chronology could be valuable preventive measures to identify crew members at increased risk of bone loss due to offloading [137].

### 4.3. Regulators of Musculoskeletal Health: A Focus on Myostatin and Irisin

Bone and muscle are known to perform an important endocrine function due to their ability to release numerous molecules, osteokines, and myokines, respectively, that promote biochemical communication between the two tissues [138]. Some of these biomarkers play important regulatory functions in the musculoskeletal system, influencing the quality of bone and muscle tissue and representing therapeutic targets for weightlessness-induced loss of bone and muscle mass. Particularly, myostatin and irisin, two important myokines released by skeletal muscle under sedentary/exertion and exercise conditions, respectively, are also known to influence bone tissue health, suggesting a significant role in bone–muscle crosstalk [139,140].

In this context, Cariati et al. evaluated the efficacy of anti-myostatin antibody treatment in primary cultures of satellite cells isolated from patients undergoing hip arthroplasty for high-energy fractures, osteoarthritis, or osteoporosis, exposed to RPM for 72 h [141]. Marked morphological changes, including cytoplasmic vacuolization of the myotubes and the presence of clear areas of necrosis, were observed in all experimental groups, especially in osteoporotic patients, in association with an increased expression of myostatin, which is probably responsible for microgravity-induced cell degeneration. Surprisingly, treatment of cells with anti-myostatin antibodies preserved cell viability by reducing the deleterious effects of RPM exposure, suggesting myostatin as a potential therapeutic target to counteract the muscle mass loss that occurs in the absence of loading and characterizes the sarcopenic condition [141]. In agreement, Smith and colleagues investigated the effects of myostatin inhibition through the administration of an anti-myostatin neutralizing antibody (YN41) in mice housed aboard the ISS, which were characterized by significant soleus muscle atrophy and marked weight loss compared to ground control mice. Noteworthy, YN41 treatment promoted an increase in body weight, lean mass, and muscle strength, with a more pronounced effect on fast-twitch muscles [142]. Similarly, inhibition of the myostatin/activin A signalling pathway, using a soluble form of the activin type IIB receptor (ACVR2B) that can bind each of these ligands, promotes significant increases in muscle and bone mass in mice housed on the ISS, with effects comparable to those seen in mice on earth [143]. Indeed, ACVR2B treatment exerted a positive effect on bone tissue, as demonstrated by the increase in long bone and vertebrae mass assessed by computed tomography (microCT), both in mice exposed to weightlessness and in mice on the ground. Furthermore, microgravity exposure and ACVR2B treatment altered several signalling pathways, inducing changes in the expression levels of key components of muscle and bone metabolism [143].

Encouraging results were also obtained using irisin, a myokine secreted by skeletal muscle in response to exercise that promotes bone formation, adipocyte browning, and cognitive gain [144,145]. The action of this myokine on bone metabolism was highlighted by Colaianni et al., who demonstrated not only that irisin promotes osteoblast differentiation in vitro, but also that osteoblasts increase ALP and COLI expression in an irisin-dependent manner [146]. Furthermore, injection of low doses of recombinant irisin (r-irisin) into young male mice is known to induce significant increases in BMD of cortical tissue, periosteal circumference, and flexural strength, indicating irisin-dependent bone stimulation [147]. Not surprisingly, irisin has been proposed as a key biomarker of weightlessness-induced bone loss, suggesting its use in counteracting microgravity- and disuse-induced musculoskeletal defects. In this regard, administration of recombinant irisin (r-irisin) in mice subjected to limb unloading preserved femoral cortical and trabecular BMD and muscle mass from atrophy [148]. In agreement, Chen and colleagues proposed that r-irisin could positively regulate osteoblast differentiation under simulated microgravity conditions by increasing the β-catenin expression and promoting the expression of osteogenic markers, such as ALP and COLI, and cell proliferation-related genes, such as cyclin-dependent kinases (CDKs) 2 and 12 and cyclins A2, D1, and E1 [149]. Finally, Sanesi et al. demonstrated the involvement of the FNDC5/irisin axis in unloading-induced bone and muscle mass loss and confirmed its potential therapeutic use in counteracting unloading-induced musculoskeletal damage [150]. Indeed, the authors analyzed the pattern of protein expression in vastus lateralis and gastrocnemius muscles and cortical bone, in parallel with serum irisin levels, in mice subjected to four weeks of hind limb unloading. Noteworthy, mice subjected to limb unloading, but treated with r-irisin, in contrast to untreated animals, retained the expression of the myosin heavy chain isoforms, MyHCIIα and MyHCIIx, as well as the anti-apoptotic factor Bcl-2, demonstrating that the bone and muscle decline occurring in mice with suspended limbs is prevented by the action of r-irisin. In addition, r-irisin was able to inhibit the expression of the senescence marker p53 and the pro-apoptotic marker Bax, confirming the role of this myokine in counteracting weightlessness-induced cell death [150]. Finally, Cariati et al. recently investigated the efficacy of a single administration of r-irisin in primary cultures of human osteoblasts exposed to RPM for three or six days [151]. Interestingly, cells treated with r-irisin during three-day RPM exposure showed significant protection from apoptotic death, in terms of cell viability, intracellular ROS production, and ratio of apoptotic markers, as well as full preservation of mineralizing capacity, as assessed by PTX3 expression. However, a single administration of r-irisin was not sufficient to counteract the damage induced by RPM exposure for a longer time, suggesting the need for repeated administration or the development of complementary strategies aimed at corroborating the action of r-irisin [151].

## 5. Conclusions

The muscle degeneration observed in astronauts during space missions is usually associated with bone mass loss. However, although weightlessness is known to cause high bone resorption and a rapid decrease in bone minerals and calcium, the underlying mechanisms are still not entirely clear, and little is known about the role of potential markers involved in microgravity exposure-induced damage. 

Much evidence has proposed altered expression of proteins and molecules involved in bone–muscle crosstalk as responsible for the impact of weightlessness on musculoskeletal health. Among these, the expression patterns of some mediators of musculoskeletal health, such as myostatin and irisin, appear to be involved in both muscle and bone damage induced by mechanical unloading and in the progression of age-related musculoskeletal disorders, such as osteoporosis and sarcopenia. However, further studies are needed not only to deepen our knowledge of the mechanisms of action of these molecules and other biomarkers, but also to identify the early sensors of mechanotransduction and to fully understand the physiological effects of weightlessness and develop strategies to counteract its detrimental consequences. Among the proposed solutions, the administration of antioxidants, regular exercise, and an appropriate nutritional approach are undoubtedly the most widely used prevention strategies. In addition, the development of experimental drug therapies, such as the use of anti-myostatin antibodies and r-irisin, are among the most innovative choices on which research has recently focused. Undoubtedly, the development of countermeasures that can prevent the bone and muscle mass loss induced by weightlessness could ensure safe space missions for astronauts and shed light on the pathogenesis of musculoskeletal disorders, highlighting the need for significant research progress in this field to address humanity’s greatest challenge.

## Data Availability

No new data were created or analyzed in this study. Data sharing is not applicable to this article.
